# Preoperative Stereotactic Radiotherapy for Spinal Metastases: Study Protocol for a Prospective Exploratory Interventional Trial

**DOI:** 10.1227/neuprac.0000000000000316

**Published:** 2026-11-12

**Authors:** Carla Marie Zwerenz, Thomas Asendorf, Bilal Younes, Torge Huckhagel, Lucas Weiser, Wolfgang Lehmann, Stefan Rieken, Jan Tobias Oelmann, Andrea Hille, Leif Hendrik Dröge, Stephanie Bendrich, Manuel Guhlich, Sandra Donath, Laura Anna Fischer, Daniela Schmitt, Veit Rohde, Rami El Shafie, Tammam Abboud

**Affiliations:** 1 Department of Radiotherapy and Radiation Oncology, University Medical Center Göttingen, Göttingen, Germany;; 2 Institute of Medical Statistics, University Medical Center Göttingen, Göttingen, Germany;; 3 Department of Neurosurgery, University Medical Center Göttingen, Göttingen, Germany;; 4 Institute of Diagnostic and Interventional Neuroradiology, University Medical Center Göttingen, Göttingen, Germany;; 5 Department of Trauma, Orthopedic and Plastic Surgery, University Medical Center Göttingen, Göttingen, Germany;; 6 Current Affiliation: Department of Radiotherapy and Radiation Oncology, University Medical Center Hamburg-Eppendorf, Hamburg, Germany

**Keywords:** Pain management, Preoperative radiotherapy, Quality of life, Spinal metastases, Stereotactic body radiotherapy, Surgical intervention, Tumor control

## Abstract

**BACKGROUND AND OBJECTIVES:**

Spinal metastases frequently require surgical stabilization and/or decompression followed by postoperative radiotherapy (RT). Preoperative stereotactic body radiotherapy (SBRT) may reduce postoperative complications and improve local tumor control. The primary objective of this exploratory trial is to evaluate safety and preliminary efficacy of preoperative SBRT in patients with spinal metastases.

**METHODS:**

The Preoperative Stereotactic Radiotherapy for Spinal Metastases study is a single-center, prospective, exploratory, interventional trial that will enroll 20 patients scheduled for surgical treatment of spinal metastases at University Medical Center Göttingen. Patients will receive SBRT (1-3 fractions) performed 1 to 3 days before surgery. The primary endpoint is local tumor control at 3 months after surgery. Secondary endpoints include pain control, wound healing disturbances, quality of life, neurological function, and instrumentation failure.

**EXPECTED OUTCOMES:**

The study is expected to provide first prospective data on the feasibility and safety of integrating preoperative SBRT into the treatment sequence for spinal metastases. It will also generate early evidence on local tumor control, perioperative morbidity, patient-reported outcomes, and instrumentation-related outcomes.

**DISCUSSION:**

Preoperative Stereotactic Radiotherapy for Spinal Metastases evaluates a novel treatment sequence intended to address limitations of postoperative RT, including delayed treatment initiation and implant-related planning challenges. The rationale is supported by preoperative SBRT approaches for brain metastases, where treatment of the intact lesion before resection has been investigated to improve target definition and avoid irradiation of postoperative cavities. The study is intended to generate initial feasibility, safety, and preliminary efficacy data that may inform the design of a subsequent randomized trial comparing preoperative with postoperative RT.

**ABBREVIATIONS:**
eCRF: electronic case report forms
PRESTRASM: Preoperative Stereotactic Radiotherapy for Spinal Metastases
RT: radiotherapy
SBRT: stereotactic body radiotherapy
SINS: Spinal Instability Neoplastic Score
UMG: University Medical Center Göttingen.

Spinal metastases occur in approximately 40% of patients with cancer, and their incidence is increasing.^1-5^ Treatment of metastatic spinal tumors typically involves a multidisciplinary strategy. Over the past 2 decades, surgery followed by radiotherapy (RT) has become an established treatment approach for spinal metastases, after a randomized trial showed that direct decompressive surgery plus postoperative RT was superior to RT alone in patients with metastatic spinal cord compression.^6^ For pathological fractures or threatening instability, treatment typically comprises instrumentation with or without decompression followed by RT to relieve pain and improve local tumor control.^7,8^

However, the conventional sequence of surgery followed by postoperative RT is associated with several practical and medical limitations. Because RT may impair postoperative wound healing, a minimum interval of 2 weeks between surgery and RT is generally recommended.^9-12^ In clinical practice, the timing of postoperative RT is therefore variable and may be delayed by wound healing disturbances or other surgery-related complications. Such delays may increase the risk of local tumor progression before RT and may further delay systemic therapy, thereby potentially contributing to poor prognosis in patients with spinal metastases.^13^

Postoperative RT may also be technically challenging because surgical implants cause dosimetric uncertainty. Implants, often made of high atomic number materials such as titanium, result in beam attenuation and scatter.^14^ In addition, some implants can cause an increase in the spinal cord dose.^15^ Another potential limitation of the postoperative approach is the theoretical risk of hematogenous dissemination of viable tumor cells during surgical manipulation of spinal metastases, which may contribute to further systemic spread and potentially affect the efficacy of postoperative RT.^16^

Preoperative stereotactic body radiotherapy (SBRT) for spinal metastases may represent a strategy to address several of these limitations. The concept of preoperative SBRT has been introduced and is currently under investigation for brain metastases.^17^ However, this concept has not been tested in the context of spinal metastases. By applying RT before surgery, treatment can be delivered to the intact tumor before postoperative anatomic changes and before placement of surgical implants. This may allow more precise target definition and may reduce the irradiated volume compared with postoperative RT, where the resection area and newly placed implants may need to be considered.

Furthermore, SBRT enables delivery of a high biologically effective dose, while shortening the overall RT treatment duration. These characteristics may contribute to improved local control, reduced treatment-related toxicity, shorter hospitalization, and a shorter interruption of systemic therapy.

## STUDY GOALS AND OBJECTIVES

The main objective of the Preoperative Stereotactic Radiotherapy for Spinal Metastases (PRESTRASM) trial is to evaluate the feasibility, safety and preliminary efficacy of a novel treatment strategy involving SBRT administered before surgical intervention for spinal metastases.

The primary objective is to assess efficacy, with local tumor control at 3 months postsurgery serving as endpoint.

Secondary outcomes include pain control, wound healing disturbances, quality of life, neurological function, instrumentation failure, and local tumor control at 6 months after treatment.

## STUDY DESIGN

The PRESTRASM study is a prospective, single-center, exploratory, single-arm interventional trial.

## METHODOLOGY

In total, 20 patients are planned to be included at University Medical Center Göttingen (UMG), Germany. Patients scheduled for surgical treatment of spinal metastases will be screened for eligibility; the main criteria are listed in Table 1. Disseminated metastases outside the spine do not preclude inclusion unless SBRT is technically infeasible or not clinically meaningful. Histological confirmation of malignant disease is required at inclusion. Recruitment will occur through the neurosurgical outpatient clinic and affiliated regional hospitals.

**TABLE 1.** Eligibility Criteria for the Preoperative Stereotactic Radiotherapy for Spinal Metastases Trial

Inclusion criteria	Exclusion criteria
1. Indication for surgical stabilization and/or decompression of a metastatic spinal tumor, as determined by the treating neurosurgeon. Eligibility is guided by the SINS score, with patients showing potential or actual instability (SINS >6)	1. Presence of motor deficits or loss of bladder and bowel control necessitating urgent surgical decompression
2. Age older than 18 y	2. Tumor extent unsuitable for SBRT (1-3 fractions) ie, cases not technically feasible within established dose constraints for the spinal cord and organs-at-risk. In practice, this applies to extensive epidural disease causing spinal cord compression or to paraspinal soft tissue components extending beyond 3 vertebral segments
3. Estimated survival >6 mo	3. Radiosensitive histology, such as germ cell tumor, lymphoma, or multiple myeloma. and/or a stable spine not requiring surgical stabilization (SINS ≤6)
4. The patient is able to understand the nature, significance and scope of the clinical study in the context of patient information and to base the decision to participate in the study on this	

SBRT, stereotactic body radiotherapy; SINS, Spinal Instability Neoplastic Score.

After written informed consent and confirmation of eligibility, patients will undergo baseline clinical assessment and CT and MRI according to core Spine Response Assessment in Neuro-Oncology recommendations.^18^ Figure summarizes the study timeline, procedures, and assessment time points.

**FIGURE.**
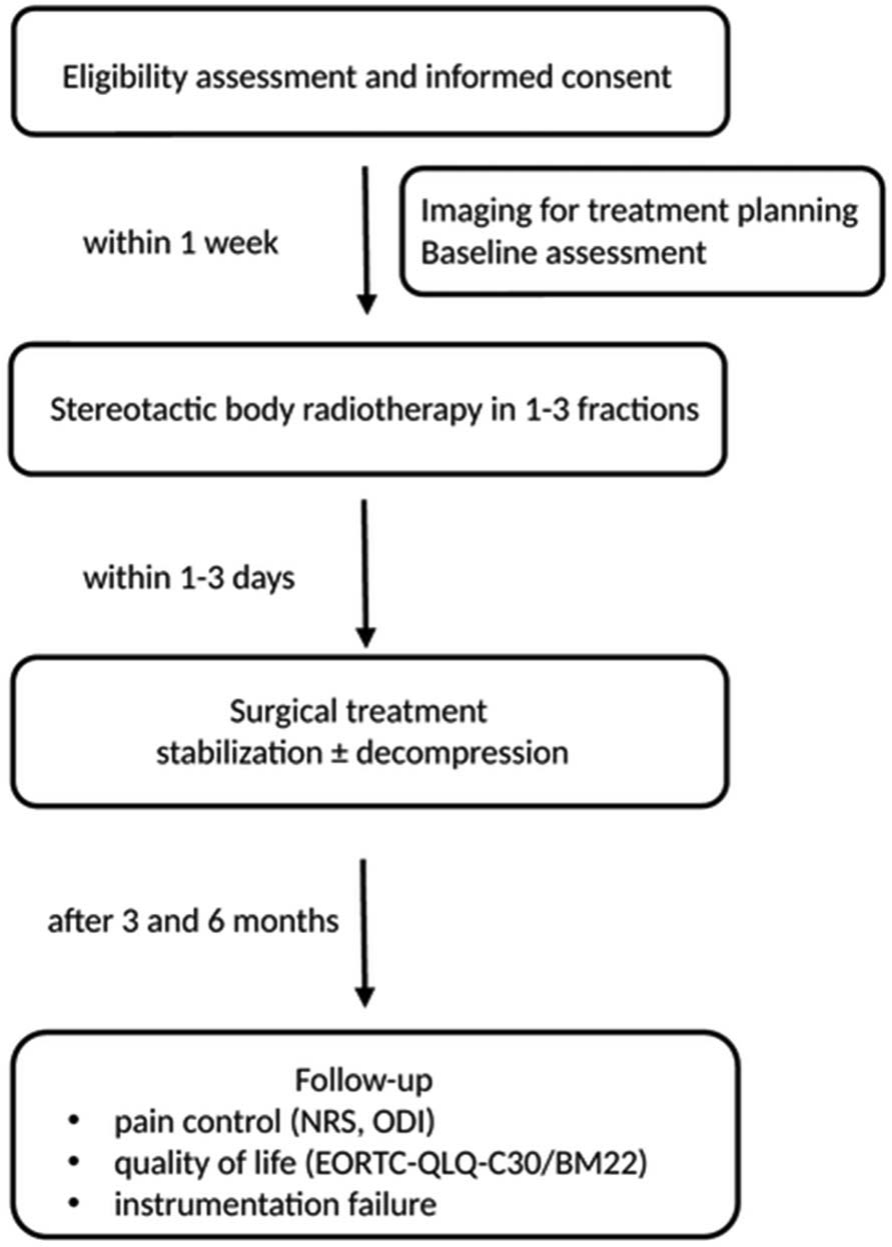
Flow chart of the Preoperative Stereotactic Radiotherapy for Spinal Metastases trial. EORTC-QLQ-C30/BM22, European Organisation for Research and Treatment of Cancer Quality of Life Questionnaire Core 30/Bone Metastases Module; NRS, Numeric Rating Scale; ODI, Oswestry Low Back Disability Questionnaire.

### SBRT

SBRT will be performed before surgical intervention and delivered in 1 to 3 fractions so as not to relevantly delay surgery. SBRT will begin within 1 week of the patient providing informed consent for trial participation. Surgical intervention is planned 1 to 3 days after completion of SBRT.

For SBRT treatment planning, patients will be immobilized according to institutional guidelines and in adherence to the European Society for Radiotherapy and Oncology clinical practice guidelines for SBRT of spine metastases.^19^ A planning CT scan with 2-mm slice thickness will be acquired in treatment position. Pretreatment MRI will be mandatory and co-registered to the planning CT for target volume delineation.

Target volume delineation will follow the principles of the International Spine Radiosurgery Consortium Consensus Guidelines for Target Volume Definition in Spinal Stereotactic Radiosurgery.^20^ Dose prescription will aim to deliver an ablative dose while respecting dose constraints for normal tissues and organs at risk. The recommendations by Emami et al, the Quantitative Analyses of Normal Tissue Effects in the Clinic (QUANTEC), and the UK Stereotactic Ablative Body Radiotherapy (UK SABR) consortium will serve as practical guidance for treatment planning.^21-23^

SBRT will be delivered as image-guided RT using pretreatment position verification and intrafractional motion management, for example stereoscopic X-rays or cone-beam CT, to ensure precise dose application. The application or discontinuation of systemic treatment in the timeframe around SBRT will be decided by the treating physician on an individual basis and according to the available evidence for the individual case.

### Neurosurgical Intervention

Surgical intervention will be performed either to decompress the spinal canal, to establish spinal stability, or to achieve both purposes. Decompression of the spinal canal is indicated when clinical or radiological signs of spinal cord or spinal nerve compression are present. The surgical technique depends on the compressed structure and usually includes laminectomy for spinal cord decompression and additional foraminotomy in cases of spinal nerve compression.

Surgical stabilization is indicated when the spinal metastasis is associated with a pathological fracture or if there is a significant risk of spinal instability according to the Spine Instability Neoplastic Score (SINS)^24^: potential instable spine (SINS 7-12) and instable spine (SINS 13-18).

Surgical technique includes dorsal instrumentation for the involved spine segments using pedicle screws. These are implanted with the help of a spinal neuronavigation system (Brainlab).

The extent and type of surgical resection, including decompression and stabilization procedures, are systematically documented and will be used in subsequent outcome and subgroup analyses. Postoperative imaging will consist of CT to assess the integrity and position of surgical implants. 

### Outcome Assessment

The primary outcome measure is local tumor control at the level of the involved spinal segments, assessed by MRI and CT at 3 months after surgery and compared with preoperative and immediate postoperative imaging. Tumor status will be categorized according to Response Evaluation Criteria in Solid Tumors, version 1.1, as local control achieved, including complete response, partial response and stable disease, or local control not achieved, defined as progressive disease.^25^

Secondary outcome measures include pain control, evaluated using the Numeric Rating Scale and the Oswestry Low Back Disability Questionnaire at baseline and during follow-up, as well as the incidence of wound healing disturbances during hospitalization and subsequent visits. Additional outcomes are health-related quality of life, assessed according to the European Organisation for Research and Treatment of Cancer Quality of Life Questionnaire Core 30 and its Bone Metastases Module. Further outcomes include the occurrence of implant or instrumentation failure, determined by radiological findings or the need for revision surgery. In addition, local tumor control at 6 months will be assessed by MRI and CT, compared with preoperative and immediate postoperative imaging. Neurological function will also be evaluated at each assessment timepoint using established clinical scales. A complete overview of scheduled assessments is displayed in Table 2.

**TABLE 2.** Schedule of Assesments (Standard Protocol Items: Recommendations for Interventional Trials Diagram)

	Before SBRT	Before surgery	After surgery	At discharge	At 3 mo follow-up	At 6 mo follow-up
Visit	1	2	3	3	4	5
Time to procedure	72-24 h before SBRT	72-24 h before surgery	24-72 h after surgery	5-10 d after surgery	80-100 d after surgery	170-190 d after surgery
Patient informed consent	X					
Inclusion/exclusion criteria	X					
Medical history	X					
Recording pain medication	X	X		X	X	
Pain rating (NRS)	X	X		X	X	X
Oswestry low back disability questionnaire	X			X	X	X
Karnofsky performance status scale	X			X	X	X
Histology				X		
MRI scan	X				X	X
CT scan	X		X		X	X
QoL Questionnaires (EORTC QLQ-C30, QLQ-BM22)	X				X	X

CT, computed tomography; EORTC QLQ-C30, European Organization for Research and Treatment of Cancer Quality of Life Questionnaire Core 30; QLQ-BM22, Quality of Life Questionnaire Bone Metastases Module; NRS, numeric rating scale; QoL, health-related quality of life; SBRT, stereotactic body radiotherapy.

## DISCUSSION

The PRESTRASM trial is designed to systematically investigate preoperative SBRT as a novel treatment concept for patients undergoing surgery for spinal metastases. The study addresses limitations of the conventional sequence of surgical stabilization and/or decompression followed by postoperative RT.

The concept of preoperative SBRT has already been explored in brain metastases^26-28^ and is currently being evaluated in prospective clinical studies.^29,30^ In spinal oncology, alternative strategies to optimize the timing of RT have included intraoperative RT combined with kyphoplasty or spinal stabilization surgery.^31,32^ PRESTRASM transfers this rationale to spinal metastases and prospectively evaluates preoperative hypofractionated SBRT followed by surgery.

The main limitation of this study is the absence of randomization and a control group. This design was chosen to ensure feasibility and to generate initial prospective data on a treatment concept that has not yet been systematically evaluated for spinal metastases. With a planned sample size of 20 patients, the study is not designed to demonstrate definitive comparative efficacy. Instead, it will provide exploratory evidence on feasibility, safety, and preliminary efficacy.

If PRESTRASM demonstrates acceptable safety and promising preliminary efficacy, the results may support the design of a future randomized phase II/III trial comparing preoperative SBRT with the current standard sequence of surgery followed by postoperative RT.

## TRIAL STATUS

The PRESTRASM trial is currently active and recruiting at UMG, Germany. The first patient was enrolled in September 2025.

## SAFETY CONSIDERATIONS

Treatment-associated toxicity will be documented by the treating physician according to the Common Terminology Criteria for Adverse Events, version 5.0.^33^ In addition, patients will independently report perceived toxicity using the patient-reported outcome version of the Common Terminology Criteria for Adverse Events.^34^

Serious adverse events are not expected. However, worsening of neurological status during RT may occur. All patients will therefore be admitted to hospital during RT and will undergo close clinical surveillance. Any signs of relevant neurological deterioration will lead to interruption of RT, and surgical intervention will be performed if clinically required.

An interim analysis will be performed after inclusion of 10 patients and will evaluate the rate of perioperative neurological deterioration in comparison with available literature.

The main drop-out criteria are withdrawal of patient consent, significant violation of the study protocol, and new motor deficits or loss of bladder or bowel control before surgery. The study may be terminated if the interim analysis after inclusion of 10 patients reveals a significantly higher rate of perioperative paresis or loss of bladder or bowel control than reported in the literature. Major unexpected events may also lead to termination of the study.

## FOLLOW-UP

Each participant will be followed for a minimum of 6 months after surgery. Postoperative assessments will be performed during the inpatient stay, at discharge, and at predefined follow-up visits 3 and 6 months after surgery.

Clinical status, pain, functional impairment, quality of life, toxicity and neurological function will be assessed using the instruments defined in the methodology section. CT and MRI will document postoperative status, implant integrity, and local tumor control.

Concomitant and adjuvant systemic therapies will be documented at baseline and during follow-up, including their timing relative to RT and surgery.

After 6-month follow-up, routine clinical care will continue, with clinical and imaging data documented in a prospective registry to monitor long-term tumor control and overall survival.

## DATA MANAGEMENT AND STATISTICAL ANALYSIS

Study data are digitally recorded using electronic case report forms (eCRF) and stored in a pseudonymized format within a REDCap® database that meets all technical Good Clinical Practice requirements. The database is securely managed, with continuous data quality checks and daily backups retained for 90 days. After publication of the primary results, an anonymized copy of the database is published to comply with findable, accessible, interoperable, reusable (FAIR) data principles.^35^

All data are pseudonymized and can only be decoded by the responsible study physicians. Third-party access is restricted to authorized personnel for quality assurance or monitoring, and only with patient consent. Personal data are stored separately from study data and are deleted if a participant withdraws consent. Study data are kept for at least 10 years after completion, after which personal data are deleted. No personal data are shared, and any data transfer for evaluation uses only pseudonymized information.

The main aim of the trial is to gain first data on the disease control rate at 3 months. According to current literature, local control rates after SBRT of spinal metastases at 3 and at 12 months range between 87% and 29%. Accordingly, when the sample size is 20, a two-sided 95% CI for a single proportion will range from 0.669 to 0.982 when the sample proportion is 0.9. The CI was calculated using the Wilson Score Method with Continuity Correction using nQuery Version 9.2.1.0.

All enrolled patients will be considered for statistical analysis. The primary analysis will be performed as a complete case analysis including patients with tumor response assessment at 3 months according to Response Evaluation Criteria in Solid Tumors, version 1.1. The local tumor control rate at 3 months will be calculated using range preserving 95% CIs. Sensitivity analyses are performed by classifying patients who dropped out before 3 months as not having achieved local control. In the case of variable time points of disease control evaluation, Kaplan-Meier curves are reported.

Secondary endpoints are analyzed descriptively summarizing mean and SD at individual time points. Estimated marginal means for individual time points are reported and displayed in a trajectory to visualize changes over time. Local tumor control and instrumentation failure at 6 months after surgery are analyzed in the same manner as the primary endpoint.

As a subgroup analysis, we aim to compare patients who underwent instrumentation only with those who underwent decompression plus instrumentation. The comparison is performed using logistic regression for outcomes evaluated at preplanned time points and few missing data and cox-regression for time-to-event outcomes. Respectively, odds ratio and hazard ratios between the 2 groups are reported with 95% CIs.

## QUALITY ASSURANCE

Quality assurance will be ensured through protocol-defined eligibility criteria, standardized treatment procedures, and structured documentation. SBRT planning and delivery will follow institutional standards and established recommendations for SBRT of spinal metastases. Individual treatment plans will be reviewed before treatment regarding target volume coverage and organs-at-risk constraints. Image-guided RT with pretreatment position verification and intrafractional motion management will be used to support accurate dose delivery.

All data will be documented in eCRF. Data completeness and plausibility will be checked continuously during the study. Protocol deviations, complications, and serious adverse events will be documented according to institutional procedures.

## EXPECTED OUTCOMES OF THE STUDY

The PRESTRASM study is expected to provide first prospective data on the feasibility, safety, and preliminary efficacy of SBRT followed by surgery in patients with spinal metastases.

The study will provide early evidence on 3-month local tumor control and perioperative safety, including neurological status, wound healing, toxicity, and instrumentation failure. In addition, the study will provide longitudinal information on pain control, functional impairment, and quality of life.

## DURATION OF THE PROJECT

The study was originally planned for approximately 2 years, including 18 months of recruitment and at least 6 months of postoperative follow-up per participant. Recruitment started in September 2025 with inclusion of the first patient and is ongoing until the planned sample size of 20 patients has been reached. Given the specialized eligibility criteria, and the exploratory nature of the study, the actual recruitment period may extend beyond the initially projected timeframe. The study duration will therefore be adapted to allow complete recruitment and protocol-defined follow-up.

Follow-up beyond 6 months will be incorporated into a prospective clinical registry to monitor long-term tumor control and overall survival as part of routine clinical care.

## PROJECT MANAGEMENT

The principal investigators will coordinate the study. The Department of Radiotherapy and Radiation Oncology and the Department of Neurosurgery are jointly responsible for the clinical and scientific conduct of the study.

Study coordination includes patient screening, scheduling of SBRT and surgery, documentation in eCRF, follow-up coordination, and interdisciplinary communication between the participating departments.

## ETHICS

The study was approved by the Ethics Committee of UMG under the application number 14/05/2024. Approval from the Federal Office for Radiation Protection in Germany was received on May 12, 2025 (ZD 3-22464/2024-263-G). All patients provide written informed consent before study participation.

Protocol Amendments: Substantive amendments will be documented and submitted to the responsible ethics committee and regulatory authority, where required, before implementation. The trial registry and affected participants will be updated as appropriate.
